# The GAG-specific branched peptide NT4 reduces angiogenesis and invasiveness of tumor cells

**DOI:** 10.1371/journal.pone.0194744

**Published:** 2018-03-22

**Authors:** Luisa Bracci, Elisabetta Mandarini, Jlenia Brunetti, Lorenzo Depau, Alessandro Pini, Lucia Terzuoli, Silvia Scali, Chiara Falciani

**Affiliations:** Department of Medical Biotechnologies, University of Siena, Siena, Italy; University of Patras, GREECE

## Abstract

Heparan sulfate proteoglycans, HSPGs, modulate major transformations of cancer cells, leading to tumor growth, invasion and metastasis. HSPGs also regulate neo-angiogenesis which prompts cancer progression and metastatic spread. A different aspect of heparin and analogs is their prominent role in the coagulation of blood. The interplay between coagulation and metastasis is being actively studied: anticoagulants such as heparin-derivatives have anticancer activity and procoagulants, such as thrombin, positively modulate proliferation, migration and invasion. The branched peptide NT4 binds to HSPGs and targets selectively cancer cells and tissues. For this, it had been extensively investigated in the last years and it proved to be efficient as chemotherapeutic and tumor tracer in *in vivo* models of cancer. We investigated the effects of the branched peptide in terms of modulation of angiogenesis and invasiveness of cancer cells. NT4 proved to have a major impact on endothelial cell proliferation, migration and tube formation, particularly when induced by FGF2 and thrombin. In addition, NT4 had important effects on aggressive tumor cells migration and invasion and it also had an anticoagulant profile.The peptide showed very interesting evidence of interference with tumor invasion pathways, offering a cue for its development as a tumor-targeting drug, and also for its use in the study of links between coagulation and tumor progression involving HSPGs.

## Introduction

The first indication that proteoglycans are involved in cancer biology dates back to 1960 when it was observed that certain carcinomas induced abnormal expression of proteoglycans in the host stroma and in the connective tissue surrounding cancer cells [1]. Recent studies elucidated glycosaminoglycan-specific roles in cancer biology, showing their direct effect on cell receptors and interactions with growth factors.

In particular, heparan sulfate proteoglycans (HSPGs) regulate cancer progression by promoting major transformations in cell phenotype, leading to tumor growth, invasion and metastasis [2]. HSPGs have a major role in all steps of tumor proliferation, dissemination and invasion [3–5]. HSPGs overexpression leads to enhanced proliferation of many types of tumor cells [6]. HSPGs are composed of a core protein which is O-glycosylated with a glycosaminoglycan, GAG, linear chain which is post-translationally modified with sulfate residues in different position and to different extents. The huge structural diversity of HSPGs allows them to interact with a variety of proteins, such as extracellular matrix (ECM) macromolecules, growth factors, chemokines, morphogens and enzymes. HSPGs interaction with the ECM initiate signaling cascades that regulate cell-cell interaction, cell motility and pathologic invasion [4]. HSPG interact with growth factors, such as fibroblast growth factors (FGFs), heparin-binding epidermal growth factor-like growth factor (HBEFG), platelet-derived growth factor (PDGF) [7] and many others. Most of the proteins that engage with HSPGs have an heparin binding site that interacts with sulfated GAG chains [4]. HSPGs are also shed from the cell membrane while maintaining the ability to bind ligands [2]. Heparan sulfate proteoglycans are also well expressed by endothelial cells, particularly those of microvessels that generate new vasculature [8]; they can also modulate this process [9]. Cell-associated HSPGs and those in the extracellular matrix (ECM) enhance angiogenesis by acting as growth factor co-receptors, whereas heparin and the shed soluble forms of HSPGs neutralize growth factors far from their receptors and consequently inhibit angiogenesis [10–11].

A different aspect of heparin and analogs is their prominent role in the coagulation of blood. The importance of clotting in cancer has been known since the nineteenth century. The interplay between coagulation and metastasis has been actively studied: anticoagulants such as heparin-derivatives have anticancer activity [12–13] and procoagulants such as thrombin positively modulate proliferation, migration and invasion [14].

Heparin arrests the coagulation cascade where thrombin is an activator, and the two molecules also have opposite effects on tumor progression. Thrombin is a serine protease with a dual function in cancer progress: 1) on one hand, by generating fibrin and activating platelets [15] and endothelial cells, it provides a matrix for new vessels, promoting distant metastases, 2) on the other hand, by binding protease-activated receptors (PARs) in malignant cells, mainly PAR1, it directly activates proliferation, migration and invasion through multiple intracellular pathways [15–17]. Thrombin also binds heparan sulfate and heparin [18].

The branched peptide NT4 binds to HSPGs, and through this binding, discriminates between cancer and healthy tissue in human surgical specimens [19–20]. It is also efficiently internalized in cancer cells and has therefore been successfully used *in vitro* and *in vivo*, conjugated with cytotoxic units, to kill cancer cells [19, 21–22], and as an *ex vivo* and *in vivo* tracer, coupled to fluorescent probes [23].

Since NT4 binds HSPGs with sub-nanomolar affinity [24], we investigated the effects of the nude peptide, in terms of modulation of invasiveness and angiogenesis, on fibroblast growth factor 2 (FGF2) and thrombin-stimulated endothelial and tumor cells. We tested NT4 in endothelial cell proliferation, migration and tube formation assays. We also evaluated NT4 modulatory effects on cancer cell invasiveness, finding very interesting evidence of reduced neo-angiogenesis and invasiveness.

## Material and methods

### Cell lines

HUVEC (human umbilical vein endothelial cells) were grown in EGM-2 [EBM-2 supplemented with 0.1% hEGF (human epidermal growth factor), 0.1% VEGF (vascular endothelial growth factor), 0.1% R3-IGF-1 (R3-insulin-like growth factor-1), 0.1% ascorbic acid, 0.04% hydrocortisone, 0.4% hFGF-β (human fibroblast growth factor), 0.1% heparin, 2% FBS (fetal bovine serum) and 0.1% GA (gentamycin, amphotericin-B)], grown on 0.1% gelatin from porcine skin, type A, in cell culture flasks and maintained at 37°C with CO_2_. MCF-7 (human breast adenocarcinoma) and MDA-MB 231 (human breast adenocarcinoma) cell lines were grown in their recommended media (DMEM for MCF-7, Leibovitz for MDA-MB 231) supplemented with 10% fetal bovine serum, 200 μg/ml glutamine, 100 μg/ml streptomycin, 60 μg/ml penicillin, and maintained at 37°C, 5% CO_2_. MDA-MB 231 were maintained at 37°C without CO_2_. All cell lines were purchased from ATCC (The Global Bioresource Center). All experiments with HUVEC were carried out between passages 2 and 6.

### Peptide synthesis

NT4 peptide was synthesized on an automated multiple synthesizer (MultiSynTech, Germany) by standard Fmoc chemistry with HBTU (O-benzotriazole-N,N,N′, N′ -tetramethyl-uronium-hexafluoro- phosphate) (MultiSyntech) and DIPEA (N,N-diisopropylethylamine) (Merck). The tetra-branched peptide was synthesized on a Fmoc4-Lys2-Lys-beta-Ala-Wang resin (Iris Biotech). Pyro-Glu-O-pentachlorophenylester (Bachem, Switzerland) was used for the last coupling step. The peptides was finally cleaved from the resin, deprotected and lyophilized. HPLC purification was performed on a C18 Jupiter column (Phenomenex). Water with 0.1% TFA (A) and methanol (B) were used as eluents. Linear gradients over 30 min were run at flow rates of 0.8 ml/min and 4 ml/min for analytical and preparatory procedures, respectively. NT4 is soluble up to a 10mg/ml concentration. The compound was also characterized on a BrukerUltraflex MALDI TOF/TOF Mass Spectrometer. NT4 (pyELYENKPRRPYIL)4K2K-beta-Ala MS: m/z calculated for C_333_H_519_N_91_O_81_ [M+ H]+: 7094.24. Found 7095.15. HPLC RT (from 80%A to 20%A) 26.63 min.

### NT4 binding to FGF2 and thrombin

NT4 binding to FGF2 and thrombin was assayed with surface plasmon resonance. All experiments were performed on a BIA T100 instrument (GE Healthcare). Binding of FGF2 and thrombin was performed on a sensor chip previously coated with NT4. Briefly, streptavidin was immobilized by standard procedures on the sensor chip and then 30 μg/mL biotinylated NT4 in HBS-EP+ (10 mM Hepes, 150 mM NaCl, 3.4 M MEDTA, 0.05% polysorbate 20, pH 7.4) was injected for 90s at the flow rate of 10 μL/min into the flow cell.

FGF2 (10 μg/mL, in HBS-EP+) and thrombin (10 IU/ml, in HBS-EP+) were injected for 120s at a flow rate of 30 μL/min onto immobilized NT4. A flow cell coated only with streptavidin was used as reference.

### Cytotoxicity to HUVEC

HUVEC were plated at a density of 8 x 10^3^ per well in 96-well pre-coated microplates with 0.1% gelatin from porcine skin, type A (Sigma Aldrich) and incubated at 37°C and 5% CO_2_. Different concentrations of NT4 peptide (50–0.195 μM) were added 24 h after plating. After 24 h, growth inhibition was assessed using a 3-(4,5-dimethylthiazol-2-yl)-2,5-diphenyltetrazolium bromide (MTT) test. Data was analyzed by nonlinear regression analysis using GraphPaD prism 5.03 software.

### Binding to HUVEC

100,000 cells/experiment were incubated in 96-well U-bottom plates for 30 minutes at room temperature with 1 μM NT4-biotin and 20, 10 or 1 μg/ml heparin in PBS-EDTA 5 mM-BSA 0.5%. Cells were then incubated with 1 μg/ml streptavidin-FITC for 30 minutes at room temperature. Flow cytometric analysis was done with 10,000 events using a BD FACSCanto II instrument (BD, NJ. USA). The results were analyzed by FCS Express 6 flow cytometry software.

### Cell proliferation

HUVEC, starved in EBM-2, were plated at a density of 2 × 10^3^ per well in 96-well pre-coated microplates with 0.1% gelatin from porcine skin, type A (Sigma Aldrich) and incubated at 37°C and 5% CO_2_.

76 pM FGF-2 (Miltenyi Biotec) and 50 μg/ml heparin (Sigma Aldrich) were added 24 h after plating, in the presence or absence of two different concentrations of NT4 peptide (10 μM and 1 μM). Cell growth was analyzed after 24 h of incubation at 37°C. Cell growth was assessed by 3-(4,5-dimethylthiazol-2-yl)-2,5-diphenyltetrazolium bromide (MTT). Data was analyzed using Graph Pad Prism 5.03 software.

### Endothelial cell migration

Cell migration was measured using an *in vitro* wound healing assay. Briefly, HUVEC (28,000 cells/side) were seeded on each side of a culture insert for live cell analysis (Ibidi, Munich, Germany). Inserts were placed in wells of a pre-coated 24-well plate (20 μg/ml human collagen IV and 10 μg/ml human plasma fibronectin (Sigma Aldrich) for 2 h at 37°C) and incubated at 37°C and 5% CO_2_ to allow cells to grow to confluence. The inserts were then removed with sterile tweezers to create a cell-free area of approximately 500 μm and the cells were treated with NT4 peptide (10 μM) in complete medium. The cells were allowed to migrate in an appropriate incubator. At time point zero and after 24 h, the wound area was observed under an inverted microscope (Zeiss Axiovert 200 microscopy) at 5x magnification and photographed with a Nikon ACT-1 Version 2.63 camera. The percentage of void area with respect to time 0 was determined using ImageJ software, Wound healing Tool Option, after 24 h, when control wells had completely filled the gap.

### Tube formation assay

Tube formation by HUVEC induced with FGF-2 and thrombin in the presence or absence of NT4 and heparin was tested on Matrigel (Corning^®^ Matrigel^®^ Growth Factor Reduced (GFR) Basement Membrane Matrix). Briefly, 1.5 x 10^4^ serum-starved HUVEC (grown for 24 h in EBM-2 serum-free medium) per well of a 96-well plate were seeded onto a Matrigel layer in EBM-2 medium. Immediately after plating, the cells were treated with 0.6 nM FGF-2 (Miltenyi Biotec), 0.5 μg/ml thrombin from human plasma (Sigma Aldrich), 50 μg/ml heparin (Sigma Aldrich) and 10 μM NT4. Tubular network structures were observed after two, three and six hours, under a Leica DMi8 inverted microscope at 10x magnification. Quantification of pseudo-capillaries was performed by ImageJ Angiogenesis Analyzer and the results were expressed as number of nodes for each treatment condition.

### Collagen degradation assay

24-well culture plates were coated with a thin-layer of chilled neutralized PureCol^™^ collagen (Sigma Aldrich) at 2.7 mg/ml in serum free Leibovitz medium and DMEM medium and incubated for 2 h at 37°C to enable fiber formation. MCF-7 and MDA-MB 231 cell lines were seeded on the collagen film (1 × 10^5^/well) and cultured for 3 days in the absence of serum at 37°C. At the end of the culture period, the remaining collagen film was exposed by removing cells by repeated treatment with PET Cell Dissociation Reagent (polyvinylpyrrolidone, EGTA and trypsin in HBS). The collagen film was then fixed with 4% PFA in PBS for 20 min at room temperature. Collagen was visualized by staining with Coomassie Brilliant Blue R250 and the images captured were analyzed using ImageJ software. The degraded area was visualized as a clear unstained zone.

### Cell invasion assay

The cell invasion assay was conducted using Transwell chamber inserts (Sarstedt, Germany). The upper chambers of 24-well cell culture inserts (8 μm pore size) were coated with chilled neutralized PureCol^™^ collagen (Sigma Aldrich) (167 μg/ml) in serum-free Leibovitz medium and dried for 2 h at 37°C. 5 × 10^4^ MDA-MB 231 cells per insert were added to the upper chambers and incubated with 0.6 nM FGF-2 (Miltenyi Biotec) and 50 μg/ml heparin (Sigma Aldrich) in the presence of NT4 peptide (10 μM). Leibovitz supplemented with 10% fetal bovine serum was added to the lower chambers. After incubating for 3 days, the non-invasive cells that remained on the upper side of the insert membranes were removed using cotton swabs. The cells that invaded the lower side of the insert membranes were fixed with PFA 4% in PBS for 15 minutes and stained with 0.1% crystal violet in 200 mM MES (2-(N-morpholino)ethanesulfonic acid) pH 6.0 for 1 h at room temperature. The stained cells were observed under a Leica DMi8 inverted microscope at 10x magnification and photographed. Images were analyses with ImageJ.

### Coagulation

Blood was drawn into a test tube containing oxalate or citrate. An excess of calcium in a phospholipid suspension was mixed into the plasma sample to reverse the effect of the anticoagulant. Celite was then added as activator, and clotting time was measured optically.

### Statistical analysis

Statistical analysis was performed using Graph Pad Prism version 5.03 (GraphPad Software, USA). P values were calculated using one-way ANOVA with Dunnett post-test for proliferation assay and one-tailed Student’s t-test for endothelial cell migration, tube formation and collagen I degradation assay, by means of Graph Pad Prism 5.03 software.

## Results

### Binding to HUVEC

**Fig 1:**
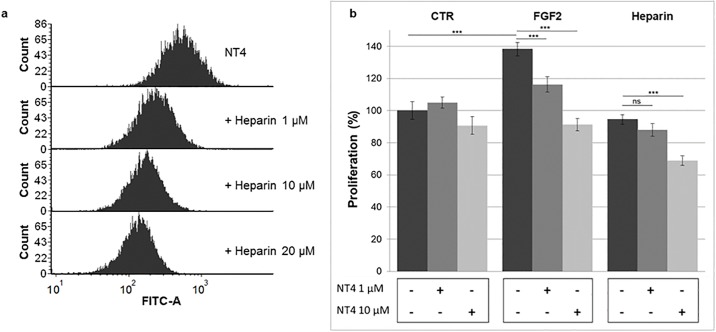
a) NT4 binding to HUVEC endothelial cells tested by cytofluorimetry: Increasing amounts of heparin displaced NT4 binding; b) Effect of NT4 on proliferation was assessed in the presence of FGF2 and heparin.

NT4 binding to HUVEC was evaluated by flow-cytometry. The cells were pre-incubated with NT4-biotin and binding was detected with Streptavidin-Atto 488. NT4 bound HUVEC and was dose-dependently displaced by heparin (Fig 1a). Toxicity of NT4 towards HUVEC was not observed at the concentrations used in the experiments (S1 Fig).

### Proliferation of HUVEC

HUVEC were cultured in EBM-2 medium. Proliferation induced by FGF2 was evaluated after 24 hours by MTT assay. FGF2 induces neovascularization, through induction of cell proliferation, migration and tube formation. We evaluated the effect of NT4 peptide on HUVEC proliferation, with and without growth factor.

HUVEC growth, as already described by others [25–26], was enhanced by nearly 40% by challenge with FGF2 (76 pM). Growth was not significantly influenced by NT4 in unstimulated HUVEC. On the contrary, when growth was triggered by FGF2, NT4 dose-dependently reduced it to control level (Fig 1b). NT4 didn’t produce any cytotoxic effect on HUVEC up to 50μM concentration in an MTT assay (S1 Fig).

The possible binding between FGF2 and NT4 was assayed by surface plasmon resonance (SPR) and no direct interaction between the two molecules was detected (S2 Fig). Binding of NT4 to the cells and not to FGF2 proved that the observed reduction in growth-factor-stimulated cell growth was a direct effect of the peptide and not mere subtraction of the growth factor from the culture medium. Cell growth was not significantly influenced by treatment with heparin but it was reduced by the combined challenge with heparin and NT4 at the higher dose.

### Endothelial cell migration

**Fig 2:**
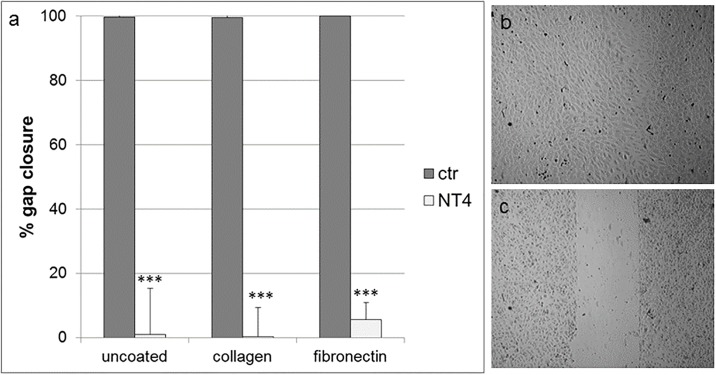
a) Effect of NT4 on migration of HUVEC on different coatings in a cell layer wound healing assay, measured as % of gap closure. HUVEC were plated on wells coated with collagen IV, fibronectin or on uncoated wells where a silicon spacer had been placed immediately before cell plating. Once cells had reached confluence, the silicon spacer was removed and cells were treated with NT4 peptide (10 μM) for 24 hours; b) Complete gap closure on fibronectin, untreated cells and C) impairment of HUVEC gap closure by NT4 on fibronectin.

In a previous study [24], NT4 dose-dependently inhibited cancer cell migration on different well coatings, such as fibronectin, collagen, matrigel and also on uncoated wells. Endothelial cell motility is a further important contributor to neoangiogenesis and tumor growth. HUVEC migration was measured using an *in vitro* wound healing assay, where cells were plated on wells coated with collagen IV, fibronectin or on uncoated wells, and a silicon spacer was placed immediately before cell plating. Once cells had reached confluence, the silicon spacer was removed and the cells were treated with NT4 peptide (10 μM) for 24 hours. Migration on the different supports was completely inhibited by NT4 (Fig 2).

### Tube formation assay

**Fig 3:**
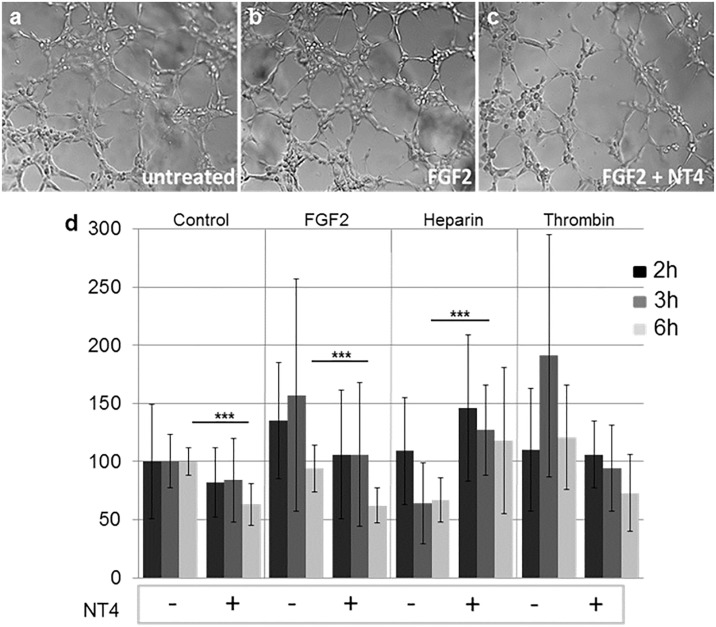
Effect of NT4 on HUVEC tube formation; a, b, c) representative images of tubes after 3 h of incubation at 37°C in the presence of FGF2 and NT4; d) variation in number of branching nodes with respect to untreated cells after 2, 3 and 6 h. Images of the wells were analyzed for the number of nodes.

The ability of HUVEC endothelial cells to form new vessels was measured in a tube formation assay, where cells are allowed to form a network on a 3D matrix. HUVEC were seeded on matrigel pre-coated wells and challenged with FGF2, heparin and thrombin, with and without 10 μM NT4. Tube formation was observed with a microscope at different time intervals and measured as number of nodes/well (Fig 3).

In the absence of NT4, FGF2 and thrombin, as expected, enhanced tube formation, and the effect was particularly evident after 3 h. NT4 inhibited tube formation in each group, particularly when the tube network was triggered by FGF2 and thrombin. Heparin, as expected [9], slightly reduced the ability of HUVEC to form tubes, whereas NT4 canceled that inhibitory effect by binding heparin.

### Collagen degradation assay

Matrix degradation is an obligatory aspect of tumor cell invasion. Metastatic cancer cells use acquired proteolytic mechanisms to penetrate basement membrane and collagen I-rich stroma, which are normally not invaded by epithelial cells [27].

MDA-MB231 are highly invasive, triple negative breast cancer cells. MCF7 are slightly invasive breast cancer cells. MCF7 and MDA-MB-231 degrade collagen differently within 72 hours. The invasiveness of these cells is at least partly due to their expression of MMPs. MMP-1, MMP-3, MMP-9 and MMP-13 are expressed by MDA-MB-231 but not by MCF7 [28]. Collagen I is a protease-dependent barrier and therefore a good model of the extracellular matrix. MCF7 and MDA-MB231 were plated on collagen type I and after 72 h of incubation, with or without NT4 and heparin, residual collagen was stained and measured.

**Fig 4:**
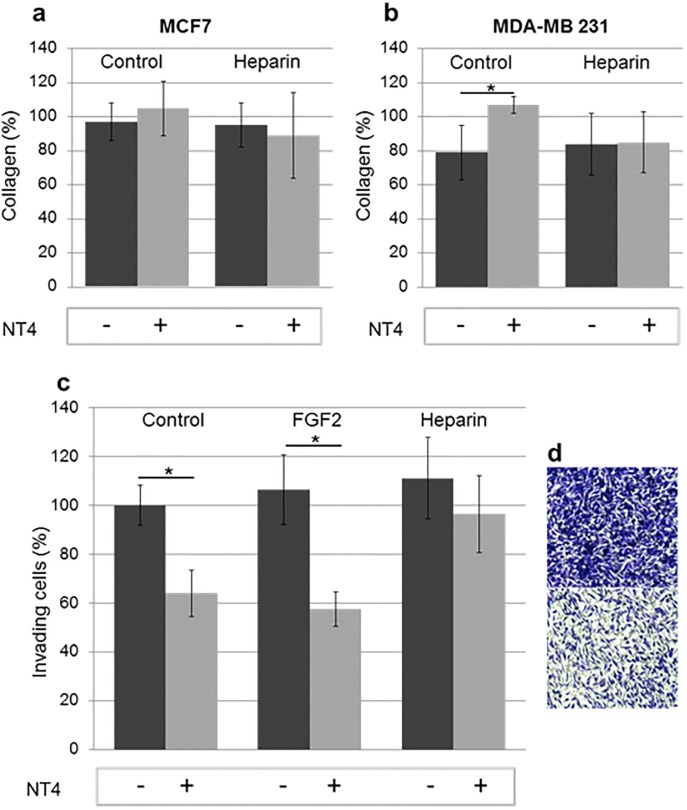
a) and b) Collagen I degradation measured after 72 h of incubation. 100% indicates intact collagen. Experiments were performed in quadruplicate; c) Collagen I invasion assay; d) MDA-MB-231 crossed a porous membrane coated with collagen (upper panel), but invasion was impaired by NT4 (10 μM) (lower panel).

MCF7 did not degrade collagen type I in the timeframe of the experiments, whereas MDA-MB-231 degraded the matrix by 20% (Fig 4a and 4b, respectively). NT4 inhibited this process. Heparin did not affect the process but interfered with NT4 inhibitory activity.

Invasiveness of cancer cells is also often assessed by invasion assay in which cell migration through a finely porous membrane coated with type-I collagen is measured. MDA-MB231 efficiently crossed the collagen I coated membrane, attracted by FBS, especially when stimulated by the presence of FGF2. When treated with 10 μ M NT4, the invasive phenotype decreased significantly (Fig 4c). MDA-MB 231 cells express the FGF2 receptor [29] and the effect of NT4 can be reasonably related to NT4 binding to GAG chains, with consequent decreased formation of the active complex FGF-2/HSPG/FGF-receptor that promotes invasion.

#### Coagulation

**Fig 5:**
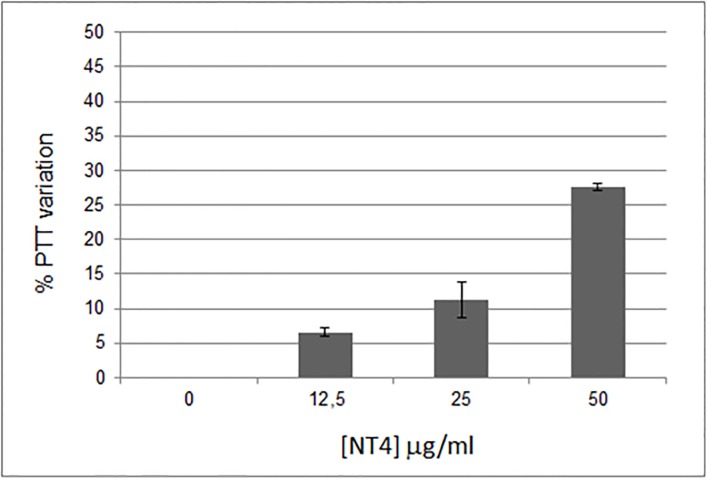
Dose-dependent anticoagulant effect of NT4.

Direct effects of NT4 on coagulation were also analyzed. Since NT4 binds heparin and is a cationic peptide with a 2+ net charge at pH 7, we expected it to have an anticoagulant effect. Coagulation activation and tumor progression are closely linked. Different components of the hemostatic system, such as thrombin and vascular cells, have an important role in neoangiogenesis and metastasis [30]. Anticoagulants oppose metastasis formation [15] and cancer-related life-threatening thrombosis events [30–31]. The effect of NT4 on partial thromboplastin time (PTT), i.e. the time it takes blood to clot, was measured. NT4 showed a dose-dependent anticoagulant effect, increasing PTT by 25% when used at a concentration of 50 μg/ml (Fig 5).

## Discussion

In cancer, HSPGs modulate critical processes, such as proliferation, migration, invasion, angiogenesis, mostly by interacting with growth factors, cytokines and growth factor receptors [4]. NT4 already proved to bind GAG chains of HSPGs [24] and in this study we observed that this binding has a major impact on endothelial cell stimulation and cancer cells invasiveness. In particular, we studied the activity of NT4 on the effects triggered by FGF2 and the heparin-thrombin pair. FGF2 is a proangiogenic factor implicated in tumor angiogenesis and already proved to induce proliferation, migration and tube formation in HUVEC [32]. Thrombin has a significant stimulatory effect on angiogenesis and induces tube formation of endothelial cells in a matrigel system [15]. Its proangiogenic effect is mediated by the upregulation of growth factors, such as VEGF, ANG-2, MMP-2 via the thrombin/PAR dependent pathway [17, 33]. Thrombin stimulates the production of specific growth factors, which in turn trigger neoangiogenesis.

In HUVEC, NT4 reduced proliferation induced by FGF2 and impaired migration on fibronectin and collagen, as well as migration on uncoated wells. NT4 also inhibited tube formation, especially when this activity was enhanced by FGF2 and thrombin. NT4 effects are likely due to the binding of NT4 with the GAG chains: this interaction prevents the formation of the GAG/growth factor/growth factor receptor complex that promotes proliferation and angiogenesis.

On the other hand, heparin reduced tube formation rates, as already described [34], while NT4 canceled this inhibitory activity. NT4 and heparin, when used in similar concentration (10 μM and 3 μM, respectively), in control groups, gave a slight decrease in proliferation of endothelial cells, almost as small as the standard deviation interval, and an increase of tube formation with respect of untreated controls. Previous studies showed that NT4 binds heparin [24] with high affinity (10^−10^ M) and since NT4 at 10 μM wasn’t pro-angiogenic nor toxic for HUVEC, the effect could be only mediated by a peptide-heparin complex, which formed at high heparin concentration (50 μg/ml, 3 μM), much higher than physiologic.

NT4 binding of HSPGs also proved to have important effects on tumor cell migration and invasion. HSPGs are known to control invasion of breast cancer cells [35]; particularly syndecan 1 and 4, upregulate the formation of FGF-2/HSPG/FGFR-1 invasion complex in MCF7 breast cancer cells [36]. In this study, MDA-MB 231 breast cancer cells were challenged to cross a finely porous membrane coated with collagen. NT4 prevented MDA-MB231 to rapidly cross the membrane in response to stimuli and also to disrupt the collagen barrier by proteolysis.

**Fig 6:**
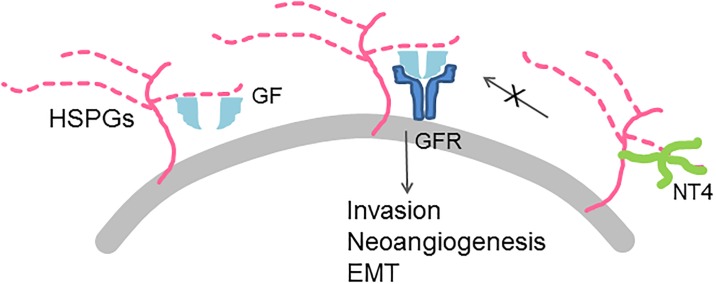
Scheme of the proposed mechanism of inhibition of invasion and neoangiogenesis by NT4.

Again, the inhibitory effect that we observe on cells migration and invasion, is likely due to the ability of NT4 to prevent the formation of the GAG/growth factor/growth factor receptor complex (Fig 6).

NT4 also showed an anticoagulant effect, which correlates with its cationic structure and may be associated with its ability to reduce a whole set of metastasis-inducing events. This effect is remarkably important in clinical practice, since inhibition of the coagulation cascade strongly suppresses tumor seeding into the blood and implantation, as well as spontaneous metastasis. It also prolongs survival and is associated with reduced risk of thrombosis in metastatic patients [30].

## Conclusions

The peptide shows very interesting evidence of interference with tumor invasion pathways, offering a cue for its development as a tumor-targeting drug, and also for its use in the study of links between coagulation and tumor progression involving HSPGs. The multi-faceted role of NT4 is most probably explained by its ability to bind HSPGs, when the latter bind growth factors. HSPGs facilitate growth factor receptor binding, which is well documented for FGF2/FGF2R [37–38]. NT4 presumably interferes with growth factor presentation to receptors by HSPGs, and most likely down-modulates receptor activation (Fig 6).

## Supporting information

S1 Fig NT4 cytotoxicity against HUVEC. (DOCX)

S2 Fig NT4 binding to FGF2 and thrombin measure by SPR. No binding is shown.
(DOCX)
