# Supplementary figures and images for: The GAG-specific branched peptide NT4 reduces angiogenesis and invasiveness of tumor cells
Source: PLoS One. 2018 Mar 22;13(3):e0194744. doi: 10.1371/journal.pone.0194744 (PMC5864057; doi:10.1371/journal.pone.0194744)

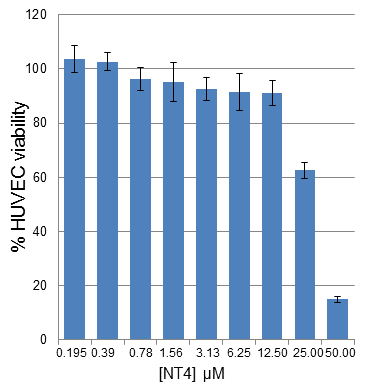


**S1 Figure**. NT4 cytotoxicity against HUVEC.

Supplement: S1 Fig — (DOCX) [file pone.0194744.s001.docx]

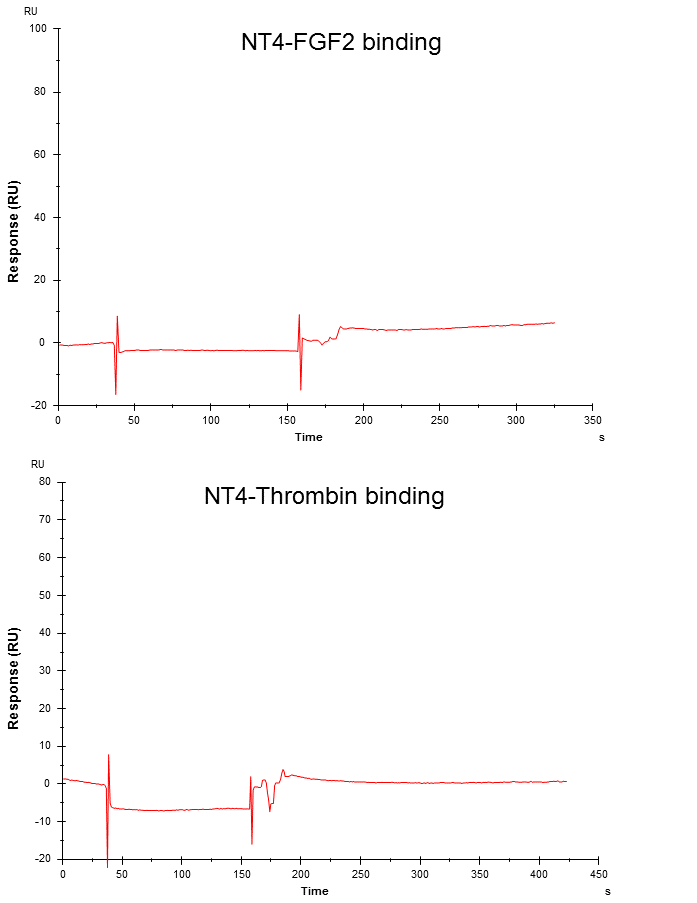


**S2 Figure**. NT4 binding to FGF2 and thrombin measure by SPR. No binding is shown.

Supplement: S2 Fig — No binding is shown. (DOCX) [file pone.0194744.s002.docx]
